# The approval of revised diagnostic criteria for DIC from the Japanese Society on Thrombosis and Hemostasis

**DOI:** 10.1186/s12959-017-0142-4

**Published:** 2017-07-03

**Authors:** Hideo Wada, Hoyu Takahashi, Toshimasa Uchiyama, Yutaka Eguchi, Kohji Okamoto, Kazuo Kawasugi, Seiji Madoiwa, Hidesaku Asakura, DIC subcommittee of the Japanese Society on Thrombosis and Hemostasis

**Affiliations:** 1 Department of Molecular and Laboratory Medicine, Mie University Graduate School of Medicine, Tsu, Mie 514-8507 Japan; 2 Department of Internal Medicine, Niigata Prefectural Kamo Hospital, 1-9-1 Aomicho, Kamo, Niigata, 959-1397 Japan; 3 Department of Laboratory Medicine, National Hospital Organization Takasaki General Medical Center, 36 Takamatsu-Cho, Takasaki, Gunma 370-0829 Japan; 4 Department of Critical and Intensive Care Medicine, Shiga University of Medical Science, Seta Tsukinowa-cho, Otsu, Shiga 520-2192 Japan; 5 Gastroenterology and Hepatology Center, Kitakyushu City Yahata Hospital, 4-18-1, Nishihon-machi, Yahatahigashi-ku, Kitakyushu, Fukuoka, 805-8534 Japan; 6 Department of Hematology, Teikyo University School of Medicine, 2-11-1 Kaga Itabashi-Ku, Tokyo, 173-8605 Japan; 7 Department of Clinical and Laboratory Medicine, Tokyo Saiseikai Central Hospital, 1-4-17, Mita, Minato-ku, Tokyo, 108-0073 Japan; 8 Department of Internal Medicine (III), Kanazawa University School of Medicine, 13-1, Takaramachi, Kanazawa, 920-8641 Japan

**Keywords:** DIC, JSTH, Diagnostic criteria, Hemostatic molecular markers

## Abstract

As proposed diagnostic criteria for DIC from the Japanese Society on Thrombosis and Hemostasis has been approved and revised, the contents and changes are informed.

Although disseminated intravascular coagulation (DIC) is a serious disease, there is no gold standard for its diagnosis, no single biomarker by which DIC can be clearly diagnosed, and no anticoagulants have been recommended for the treatment of DIC in worldwide [1]. We reviewed the diagnostic criteria for DIC including the newly proposed diagnostic criteria for DIC from the Japanese Society on Thrombosis and Hemostasis (JSTH) [2, 3]. Three diagnostic criteria for DIC have been established by the Japanese Ministry of Health and Welfare (JMHW) [4], the International Society of Thrombosis and Haemostasis [5] and the Japanese Association for Acute Medicine [6]. The three diagnostic criteria involve a scoring system based on the results of global coagulation tests (GCTs) such as the platelet count, prothrombin time (PT), fibrinogen and fibrin-related markers. Thus, there were no significant differences in the usefulness among three different diagnostic criteria for DIC [7].

For this reason, the JSTH proposed a provisional draft of the DIC diagnostic criteria, which used the classifications of “hematopoietic disorder type” which omitted the platelet count score, “infectious type” which omitted the fibrinogen score, and “basic type” based on the underlying pathology [2, 3]. An additional point was added to the GCTs scoring system if the platelet count decreased with time, and molecular markers and the antithrombin (AT) activity were added to the new criteria. To protect against misdiagnosis, 3 points were deducted if a patient had liver failure.

After the drafting of the proposed criteria [3], several evaluations were carried out to examine the issues associated with the diagnosis of the three types of DIC [8–10]. 1) What is the most useful combination for diagnosing DIC? The combination of GCTs, decreased platelet count, AT, increased soluble fibrin (SF), thrombin AT complex (TAT) or prothrombin complex F1 + 2 (F1 + 2) scores had the highest area under the curve (ARC) values and odds ratio in a analysis [8–10]. 2) Can this scoring system diagnose early-phase DIC? The JSTH diagnostic criteria could diagnose DIC several days before its onset (as defined by the JMHW criteria). 3) What score is appropriate for making a diagnosis of DIC? The ROC analysis revealed that a score of 4 points was an adequate cutoff value for hematopoietic disorder-type DIC {area under the curve (AUC) 0.979; sensitivity 97.2%; specificity 96.0%; positive predictive value (PPV) 95.4; negative predictive value (NPV) 97.6% and odd’s ratio 832} [8], while a score of 5 points (instead of the 6 points in the previous criteria) [2, 3] was suitable for the diagnosis of infectious-type DIC {AUC 0.984; sensitivity 93.7%; specificity 97.8%; positive predictive value (PPV) 98.3; negative predictive value (NPV) 91.7% and odd’s ratio 649} [9] and a score of 6 points was suitable for the diagnosis of basic-type DIC [10] {AUC 0.987; sensitivity 98.4%; specificity 94.8%; positive predictive value (PPV) 91.2; negative predictive value (NPV) 99.1% and odd’s ratio 1137}. 4) Can these diagnostic criteria predict a poor outcome? Although these diagnostic criteria can predict a poor outcome (odds ratio 2–3), there were no significant differences in the odds ratios of any of the combinations [8–11]. Thus, these diagnostic criteria have been approved as JSTH diagnostic criteria (Table 1) [12].

**Table 1:** JSTH’s DIC diagnostic criteria

	Classification of type	Basic		Hematopoietic disorder		Infectious	
GCTs	Platelet count (×10^3^/μl)	>120	0 p			>120	0 p
		80 < − ≤ 120	1 p			80 < − ≤ 120	1 p
		50 < − ≤ 80	2 p			50 < − ≤ 80	2 p
		≤50	3 p			≤50	3 p
		≥30% decrease w/in 24 h (*1)	+1 p			≥30% decrease w/in 24 h (*1)	+1 p
	FDP (μg/ml)	<10	0 p	<10	0 p	<10	0 p
		10 ≤ − < 20	1 p	10 ≤ − < 20	1 p	10 ≤ − < 20	1 p
		20 ≤ − < 40	2 p	20 ≤ − < 40	2 p	20 ≤ − < 40	2 p
		≥40	3 p	≥40	3 p	≥40	3 p
	Fibrinogen (mg/dl)	>150	0 p	>150	0 p		
		100 < − ≤ 150	1 p	100 < − ≤ 150	1 p		
		≤100	2 p	≤100	2 p		
	Prothrombin time ratio	<1.25	0 p	<1.25	0 p	<1.25	0 p
		1.25 ≤ − < 1.67	1 p	1.25 ≤ − < 1.67	1 p	1.25 ≤ − < 1.67	1 p
		≥1.67	2 p	≥1.67	2 p	≥1.67	2 p
HMMs	Antithrombin (%)	>70	0 p	>70	0 p	>70	0 p
		≤70	1 p	≤70	1 p	≤70	1 p
	TAT, SF or F_1+2_	<2-fold of normal upper limit	0 p	<2-fold of normal upper limit	0 p	<2-fold of normal upper limit	0 p
		≥2-fold of normal upper limit	1 p	≥2-fold of normal upper limit	1 p	≥2-fold of normal upper limit	1 p
Liver failure (*2)		No	0 p	No	0 p	No	0 p
		Yes	−3 p	Yes	−3 p	Yes	−3 p
DIC diagnosis		≥6 p		≥4 p		≥5 p	

*Abbreviations*: *p* points, *GCT*:global coagulation tests, *HMMs* hemostatic molecular markers

(*1): For a platelet count of >50 × 10^3^/μL, points will be added if the time-course conditions of decrease are met (no points will be added for a platelet count of ≤50 × 10^3^/μL). The maximum score for the platelet count is 3 points.

For institutions that do not measure FDP (institutions that measure only D-dimer), 1 point will be added if D-dimer increases ≥2-fold the normal upper limit. The upper limit of D-dimer is different among various D-dimer kits. However, in principle, FDP should also be measured and re-evaluation performed after the results are in hand. Fibrinogen levels are usually measured using thrombin time method in Japan.

DIC may be excluded in case within normal range of FDP or D-dimer.

Prothrombin time ratio: If ISI is close to 1.0, INR will also be acceptable (However, there is no evidence supporting recommendation of the use of PT-INR for diagnosis of DIC.). For determination of PT ratio, it is recommended to use normal pooled plasma.

DIC may be excluded in case with elevated prothrombin time ratio due to vitamin K deficiency.

Thrombin-antithrombin complex (TAT), soluble fibrin (SF), prothrombin fragment 1 + 2 (F_1+2_): For blood sampling in difficult cases and route blood sampling, false-high values may increase. Thus, in comparison with elevation of FDP and/or D-dimer, re-testing should be done if TAT and/or SF is markedly elevated. Confirmation is needed even if the results on the same day are not in time. Normal reference range is 56–213 pmol/L in F_1+2_, 0–3.2 μg/ml in SF and 0.3–1.5 ng/ml in TAT in Mie University Hospital.

Regardless of the presence or absence of DIC immediately after surgery, changes in DIC-like markers such as elevation of TAT, SF, FDP, or D-dimer or a decrease in AT, may be observed, and judgment should be made with care.

(*2) Liver failure: Corresponds to “a prothrombin time activity of ≤40% or an INR value of ≥1.5 due to severe liver dysfunction seen within 8 weeks of onset of initial symptoms following liver impairment that develops in a normal liver or a liver that is thought to exhibit normal liver function” (acute liver failure) or “cirrhosis with a Child-Pugh classification of B or C (≥7 points)” (chronic liver failure) that may be viral or autoimmune in origin, drug-induced, or caused by circulatory failure.”

Even when DIC is strongly suspected but these diagnostic criteria are not met, there should be no interference with anti-coagulation therapy based on the physician’s judgment, but repeated evaluation is necessary

In conclusion, the JSTH diagnostic criteria for DIC have been revised and approved: Infectious-type DIC is now diagnosed based on a score of 5 points instead of 6 points.

## Abbreviations

ARC: Area under the curve
AT: AntithrombinSF Soluble fibrin
DIC: Disseminated intravascular coagulation
F_1+2_: Prothrombin fragment 1 + 2
GCTs: Global coagulation tests
JMHW: Japanese Ministry of Health and Welfare
JSTH: Japanese Society on Thrombosis and Hemostasis
PT: Prothrombin time
ROC: Receiver operating characteristic curve
TAT: Thrombin-antithrombin complex
